# Attrition from Care Among Men Initiating ART in Male-Only Clinics Compared with Men in General Primary Healthcare Clinics in Khayelitsha, South Africa: A Matched Propensity Score Analysis

**DOI:** 10.1007/s10461-022-03772-9

**Published:** 2022-07-31

**Authors:** Tali Cassidy, Morna Cornell, Bubele Makeleni, C. Robert Horsburgh, Laura Trivino Duran, Virginia de Azevedo, Andrew Boulle, Matthew P. Fox

**Affiliations:** 1 Médecins Sans Frontières, 8 Mzala Street, 7784 Khayelitsha, Cape Town, South Africa; 2 Division of Public Health Medicine, School of Public Health and Family Medicine, University of Cape Town, Cape Town, South Africa; 3 Department of Epidemiology, Boston University School of Public Health, Boston, USA; 4 Centre for Infectious Disease Epidemiology & Research, School of Public Health and Family Medicine, University of Cape Town, Cape Town, South Africa; 5 Section of Infectious Diseases, Department of Medicine, Boston University School of Medicine, Boston, USA; 6 Department of Global Health, Boston University School of Public Health, Boston, USA; 7 City Health Department, Cape Town, South Africa; 8 Western Cape Provincial Department of Health, Cape Town, South Africa; 9 Health Economics and Epidemiology Research Office, Department of Internal Medicine, School of Clinical Medicine, Faculty of Health Sciences, University of the Witwatersrand, Johannesburg, South Africa

**Keywords:** HIV, Antiretroviral therapy, Retention in care, Males, Differentiated service delivery

## Abstract

Men have higher rates of attrition from antiretroviral therapy (ART) programs than women. In Khayelitsha, a high HIV prevalence area in South Africa, two public sector primary healthcare clinics offer services, including HIV testing and treatment, exclusively to men. We compared attrition from ART care among men initiating ART at these clinics with male attrition in six general primary healthcare clinics in Khayelitsha. We described baseline characteristics of patients initiating ART at the male and general clinics from 1 January 2014 to 31 March 2018. We used exposure propensity scores (generated based on baseline health and age) to match male clinic patients 1:1 to males at other clinics. The association between attrition (death or loss to follow-up, defined as no visits for nine months) and clinic type was estimated using Cox proportional hazards regression. Follow-up time began at ART initiation and ended at attrition, clinic transfer, or dataset closure. Before matching, patients from male clinics (n = 784) were younger than males from general clinics (n = 2726), median age: 31.2 vs 35.5 years. Those initiating at male clinics had higher median CD4 counts at ART initiation [Male Clinic 1: 329 (IQR 210–431), Male Clinic 2: 364 (IQR 260–536), general clinics 258 (IQR 145–398), cells/mm^3^]. In the matched analysis (1451 person-years, 1568 patients) patients initiating ART at male clinics had lower attrition (HR 0.71; 95% CI 0.60–0.85). In separate analyses for each of the two male clinics, only the more established male clinic showed a protective effect. Male-only clinics reached younger, healthier men, and had lower ART attrition than general services. These findings support clinic-specific adaptations to create more male-friendly environments.

**Supplementary Information:**

The online version contains supplementary material available at 10.1007/s10461-022-03772-9.

## Introduction

In most African settings, men are less likely to get tested for HIV, link to HIV care or initiate antiretroviral therapy (ART) than women [1–7]. Those who do are more likely to present to clinics later, with more advanced disease and have worse clinical outcomes [3–5, 8–17]. A meta-analysis found that among 20-year olds starting ART in low and middle-income countries, males on average live 22.9 more years (95% CI 18.4–27.5 years), compared to 33.0 years (95% CI 30.4–35.6 years) for females [18]. Multiple studies have shown men to have higher rates of attrition from HIV care programs than women [5, 8, 19–31]. In South Africa, ART coverage is substantially lower among adult males (58%) compared with adult females (64%) [32]. In 2017, 36% of the nearly 7 million HIV-positive adults living in South Africa were male, but males accounted for 52% of AIDS deaths [32]. Being on ART not only reduces mortality, but also decreases morbidity and transmission [33–35].

Explanations for men’s low attendance and poor outcomes include public health systems that are historically built around maternal and child health, systematic under-funding of men’s services compared to women’s [4, 13, 36, 37], and notions of masculinity that are at odds with both illness [38, 39] and expected patient behavior [40]. Despite higher attrition among males on ART, few strategies have been developed to specifically address poor HIV outcomes for males in low and middle-income countries with generalized epidemics [12, 13, 36].

In 2014, in response to low male engagement and retention in care, the City of Cape Town’s health department, supported by Médecins Sans Frontières started a male clinic, (“Male Clinic 1”) [41] and in 2016 a second male clinic opened Both clinics are located in Khayelitsha, a high HIV prevalence, high-poverty peri-urban area in Cape Town [42, 43]. We evaluate this intervention by comparing attrition from care among men at these two male-only clinics to men attending six general primary healthcare clinics in Khayelitsha.

## Methods

### Study Design

We conducted a propensity score matched cohort study of adult males receiving ART at primary care clinics in Khayelitsha, comparing attrition among males in male-only clinics to males in general clinics.

### Intervention

Male Clinic 1 was opened in 2014 by the City of Cape Town’s health department, supported by Médecins Sans Frontières. The clinic is located near a transport hub and on its own premises, but not far from a larger health center. This service is staffed exclusively by males, is promoted as a males-only space, and offers HIV testing and counselling, ART initiation and dispensing, sexually transmitted infection (STI) diagnosis and treatment, and other primary healthcare services [44]. In July 2016, another clinic following the same model (“Male Clinic 2”) began offering ART services at a small clinic above a transport hub.

### Population and Setting

The City of Cape Town’s Health department offers HIV care at 10 primary healthcare clinics in Khayelitsha, including two male-only clinics, six general primary healthcare clinics (with no particular male-targeted programs), and two youth-targeted clinics. Department of Health clinicians provide free services at all facilities, with support from non-governmental organizations who manage all lay adherence counselors at each facility. For the time period considered in this paper, Médecins Sans Frontières paid for and managed the counselor at Male Clinic 1, but not Male Clinic 2.

The study population consisted of adult (18 years and older) males who first initiated ART between 1 January 2014 (the year that Male Clinic 1 opened) and 1 April 2018 at the two male-only clinics or six general primary healthcare clinics. If individuals were not ART naïve or transferred into the clinic (as recorded by clinic) they were excluded. Youth-targeted clinics were excluded from the analysis as these clinics represent a separate novel model of targeted care, and do not provide care to men over the age of 25. Patients who had tuberculosis at ART initiation were excluded because these patients were often referred out of the male clinics for management at the general clinics. We excluded patients known to have initiated ART on a regimen not containing tenofovir (TDF) or efavirenz (EFV) as patients requiring non-tenofovir or non-efavirenz regimens at male clinics were generally referred to a general clinic.

### Data Source

All data for this study came from routine patient data collected from patient folders at City of Cape Town clinics in Khayelitsha. Data capturers at each clinic capture data electronically from the patient folder after each patient ART visit. Data includes patient demographics, visits, regimens dispensed, laboratory results, and events such as deaths or transfers.

### Measures and Follow-Up Time

The exposure of interest was receiving HIV treatment at one of two male clinics compared with receiving care at a general clinic. Our primary outcome was attrition from HIV care, a measure including all patients who were lost to follow-up or who died. Death was passively reported by families so it was assumed that, as reported in similar contexts, some patients who had died would have been misclassified as lost to follow-up in clinics [45–47]. A patient was considered lost to follow-up the first time there was a nine month gap in care, even if he later returned to care. Patient visits are generally 2–4 months apart, so a nine-month gap in care represents approximately six months with no ART. The date of last visit before the gap in care was used as the outcome date [48]. In the exploratory analysis, 6- and 12-month attrition was defined as having the outcome of attrition from HIV care, and having the last visit before attrition less that 6 months (in the case of 6-month attrition) or 12 months (in the case of 12-month attrition)after ART initiation.

The dataset was closed on 30 September 2018. Follow-up time for each person started on the date of ART initiation and ended on the last visit date before dataset closure, regardless of their outcome. Patients who transferred out were censored on their last visit date at their original clinic, even if the transfer was to another clinic represented in this dataset. If a patient’s last attended visit was the same as their ART initiation date, one day of follow-up was added to prevent them from being excluded from Kaplan–Meier and Cox proportional hazards analyses [48]. Baseline CD4 count was defined as the closest available result to the date of ART initiation, between 180 days before and 30 days after initiation.

### Analysis

#### Multiple Imputation

Chained multiple imputation [49–51] was performed using the ice procedure in Stata [52, 53] for missing values of baseline WHO disease stage and CD4 count. Missing data were assumed to be missing at random. We created 20 imputed datasets (see Table 4 and 5 in Supplementary file 2).

#### Descriptive Analyses

Predefined potential confounders included indicators of baseline health, date of ART initiation, and age, variables known to be associated with ART outcomes [17, 19, 20, 54], and plausibly also associated with clinic type. We describe these covariates, stratified by clinic type, before and after propensity score matching. We also describe attrition by 6 and 12 months, stratified by covariates, for observations with at least 9 and 15 months of follow-up time respectively, excluding those who transferred out before 6 and 12 months respectively.

#### Propensity Score Matching

We used a matched exposure propensity score approach to control for confounding. Propensity scores were generated using logistic regression, with clinic type (male clinics vs general clinics) as the dependent variable. Independent variables were baseline WHO stage, age, year of ART initiation and CD4 count, which were selected from the predefined potential confounders based on observed associations with both attrition and male clinics in the descriptive analyses. This process was repeated for each of the imputed datasets and an average propensity score for each observation was calculated across datasets [55]. The exposed were matched, in random order, 1:1 to the unexposed patient with the closest average propensity score (nearest neighbor matching), without replacement [56].

#### Time-to-Event Analyses

We performed a Cox proportional hazards regression on the matched data to estimate the hazards ratio (HR) of attrition associated with attending male clinics compared to general clinics. Where appropriate the results of the analyses from the multiple imputed datasets were combined using Stata’s *mi estimate* feature, which uses Rubin’s methods [57] to adjust coefficients and standard errors for the variability between imputations. To adjust for any residual confounding after matching on propensity score, covariates were individually added to the model to observe their effect on the HR but none were retained as none met the threshold of changing the HR by more than 10% (see Table 2 in Supplementary file 2). To support the Cox regression, and for descriptive purposes, Kaplan–Meier curves of each male clinic cohort and their matched controls, with attrition as the outcome, are also presented.

Goodness of fit tests using Schoenfeld residuals suggested no evidence of violation of the proportional hazards assumption (p = 0.95, see Table 3 in Supplementary file 2). For the survival curves and model diagnostics, only the first imputed dataset was used, because after propensity score generation and matching clinic type, outcome and outcome date were identical across datasets.

#### Sensitivity Analyses

We performed several sensitivity analyses of the Cox regression analysis. First, the analysis, including propensity score matching procedures, was performed separately for each male clinic, to see if there was a difference in effect between the two clinics. We did an additional analysis which excluded Male Clinic 1 patients and follow-up time after the first two years of the clinic’s ART services, to see if any differences between the two male clinics could be explained by Male Clinic 1 being a more established clinic. Second, we changed the outcome definition to reflect the patient’s status at dataset closure. The clinic data system automatically classified patients as lost to follow-up if a patient was three months late for a scheduled visit with no subsequent visit. If a patient returned to care before dataset closure they were no longer considered lost to follow-up. Third, we considered the effects of ‘silent transfers’ [58, 59], where patients switched to another facility but were not captured as transfer-outs. We manually searched for results from patients who were lost to follow-up on the National Health Laboratory System, which contains laboratory results from all patients in the public sector, regardless of where they attend care. Patients were reclassified as transfers if they had a viral load (a routine HIV monitoring test) result within one year of their last known visit, at any primary healthcare facility. We excluded blood tests that were performed at tertiary care facilities as these were likely done because the patient presented with an illness, and did not necessarily indicate that the patient was in ART care.

#### Quantitative Bias Analysis

A hypothesized cluster of behavioural characteristics, including mental health and attitudes towards healthcare and female healthcare workers, may be associated with male clinic attendance and attrition [60–62]. Considering this cluster of characteristics as a single confounder, we conducted a quantitative bias analysis. We hypothesized plausible distributions of (1) confounder prevalence in general clinics (triangular distribution 0–20%, mode = 10%); (2) association between the confounder and male clinic attendance (triangular distribution 1.5–2.5, mode = 2), and (3) association between the confounder and attrition at any time (triangular distribution 0.5 to 0.9, mode = 0.7). Sampling from these distributions, we ran 100,000 simulated analyses where HRs were adjusted for the unmeasured confounder. The resulting HRs show the effect of male clinics on attrition that incorporate hypothesized systematic and random error [63]. The median bias-adjusted HR is presented, along with a 95% interval (2.5–97.5 percentile) (See Supplementary file 1 for additional details).

### Ethics

Ethics approval was granted by the University of Cape Town’s Human Research Ethics Committee (HREC395/2005), who waived consent as the analyses used de-identified data collected as part of routine patient care.

## Results

**Fig. 1:**
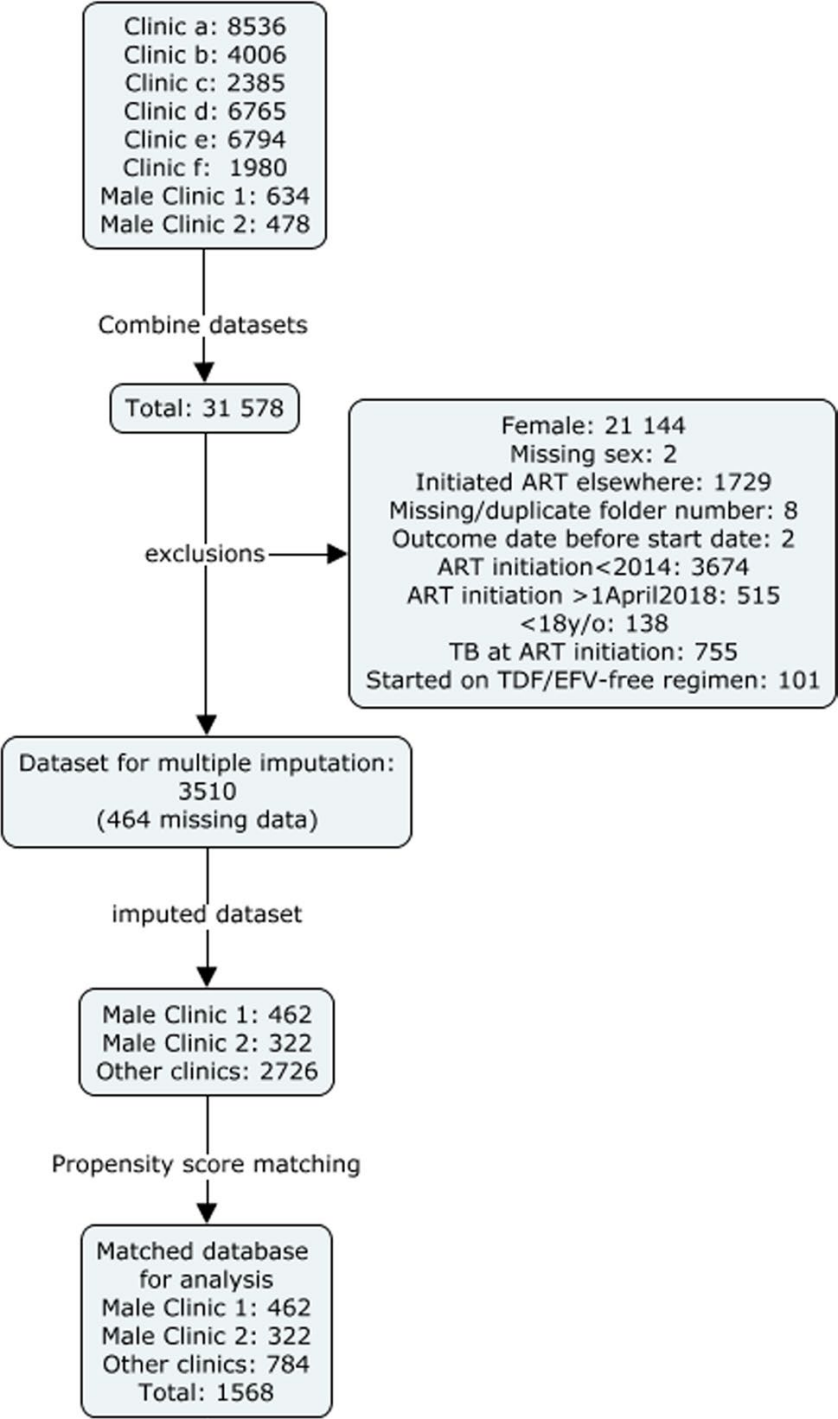
Flow chart for inclusion in analysis

We had data for 31 578 patients ever on ART in male-targeted or general primary healthcare clinics run by City Health in Khayelitsha. After exclusions, the analytic dataset included 3510 observations: 462 observations from Male Clinic 1, 322 from Male Clinic 2, and 2726 eligible matched comparisons from other clinics (Fig. 1).

Patients at Male Clinic 2 were most likely to have initiated ART recently, with 89% of patients initiating after ART eligibility criteria were removed in 2016, compared to 39% in the general clinics (Table 1). Men initiating ART at male clinics were younger than those in the general clinics [31.2 (IQR 26.9–36) compared to 35.5 (IQR 30.3–42.2) years old]. General clinics had a higher proportion of patients initiating ART at WHO disease stages 2–4 (31%) compared to Male Clinic 1 (7%) and Male Clinic 2 (2%). Men initiating treatment in the male clinics had a higher median CD4 count than general clinics [Male Clinic 1: 329 (IQR 210–431), Male Clinic 2: 364 (IQR 260–536) vs general clinics 258 (IQR 145–398) cells/mm^3^]. General clinics had a higher proportion of missing data on disease stage at ART initiation and CD4 count (Table 1).

**Table 1:** Distribution of covariates by clinic group, before and after imputation and propensity score matching

	Before multiple imputation and propensity score matching			After multiple imputation and propensity score matching	
	Male Clinic 1	Male Clinic 2	General clinics	Male clinics	General clinics
N	462	322	2726	784	784
Year of ART initiation					
2014	9%	0%	19%	5%	6%
2015	27%	0%	25%	16%	14%
2016	29%	25%	26%	27%	27%
2017	27%	58%	25%	40%	41%
2018	8%	17%	6%	11%	11%
After 1 Sept 2016^a^	44%	89%	39%	63%	60%
Age					
18–24	15%	15%	7%	15%	14%
25–34	55%	59%	41%	57%	57%
35 +	30%	26%	52%	28%	29%
Median age (years) (IQR)	31.6 (27.4–36.9)	30.7 (26.6–35.3)	35.5 (30.3–42.2)	31.2 (26.9–36)	31.3 (27.1–36.2)
Baseline WHO stage					
Stage 1	89%	93%	59%	95%	91%
Stage 2	4%	2%	16%	3%	6%
Stage 3	3%	0%	12%	2%	3%
Stage 4	1%	0%	3%	1%	0%
Stage 2–4	7%	2%	31%	5%	9%
Missing	3%	4%	10%	–	–
Baseline CD4 count (cells/mm^3^)					
< 200	22%	11%	34%	19%	26%
200–349	32%	32%	28%	33%	26%
350–500	32%	25%	19%	30%	26%
> 500	13%	26%	11%	19%	22%
Missing	1%	5%	8%	-	-
Median CD4 count (IQR)	329 (210–431)	364 (260–536)	258 (145–398)	343 (233–455)	335 (193–484)

^a^On 1 September 2016 CD4 thresholds were removed as eligibility requirements for ART initiation, allowing anyone that tested positive to initiate ART immediately

Descriptive analyses before imputation or propensity score matching revealed that Male Clinic 2 had a higher risk of 12-month attrition (35%) compared to Male Clinic 1 (23%) or general clinics (32%) (Table 2). Risk of attrition by six and 12 months was associated with more recent ART initiation and younger age. Baseline WHO stage was not associated with attrition, but missing information was associated with higher attrition and those healthier at baseline were at a slightly lower risk of attrition. We observed 31% attrition at 12 months for those with a CD4 count below 200 cells/mm^3^, compared to 33% for those with CD4 count above 500 cells/mm^3^. For survival estimates at months 6 and 12 by covariates see Table 1 in Supplementary file 2.

**Table 2:** Attrition from Care at 6 and 12 months by covariates (full dataset before imputation or propensity score matching)

	Attrition by 6 months N = 3103^a^	Attrition by 12 months N = 2593^b^
Total	23%	31%
Clinic		
General clinics	23%	32%
Male clinic 1	18%	23%
Male clinic 2	28%	35%
Initiating in year		
2014	22%	30%
2015	21%	29%
2016	23%	32%
2017	26%	35%
Guidelines CD4 < 350 (before 2015)	22%	30%
Guidelines CD4 < 500 (1 Jan 2015–31 Aug 2016)	21%	30%
After Universal Test and Treat (1 Sept 2016)	26%	34%
Age		
18–25 years	26%	36%
25–35 years	25%	34%
35 + years	21%	28%
WHO stage at initiation		
Stage 1	22%	30%
Stage 2	24%	31%
Stage 3	25%	35%
Stage 4	19%	26%
Stage 2–4	24%	32%
Stage missing	28%	38%
Baseline CD4 Count		
< 200	22%	31%
200–350	22%	30%
350–500	24%	31%
> 500	25%	33%
CD4 count missing	30%	36%

^a^6-Month attrition is defined as a gap of more than 9 months with last visit before gap occuring before month 6 and is only presented for those who initiate ART more than nine months before dataset closure. Those who transferred out < 6 months after initiation were excluded

^b^12-Month attrition is defined as a gap of more than 9 months with last visit before gap occuring before month 12 and is only presented for those who initiate ART more than 15 months before dataset closure. Those who transferred out < 12 months after initiation were excluded

### Propensity Score Estimation and Matching

For males clinics patients, the median propensity score predicting the probability of attending a male clinic was 0.35 (IQR 0.23–0.46), compared to 0.16 (IQR 0.06–0.28) among general patients (Supplementary file 2, Fig. 1). After matching, the median propensity score was 0.35 for both male clinics (IQR 0.23–0.46) and general clinics (IQR 0.23–0.43).

Propensity score matching reduced the association between exposure and covariates, but there were still small differences in terms of ART start date, age, disease stage and CD4 count at initiation (Table 1 and Supplementary file 2, Fig. 2).

Compared to males that were not matched to a male clinic patient, matched general clinic patients initiated ART at later dates, younger age, higher CD4 counts and less advanced disease stage (see also Fig. 2 in Supplementary file 2).

### Analysis of Matched Cohort

The matched cohort (N = 1568) represented a total of 1568.9 person-years, 807.6 of which were in the general clinics and 761.3 of which were in the male clinics.

**Fig. 2:**
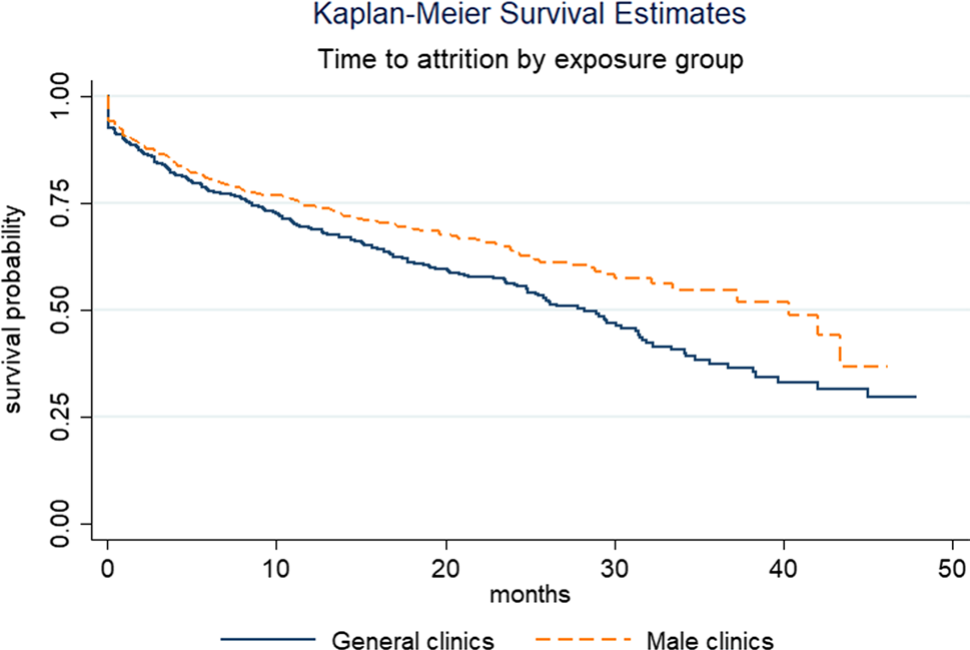
Kaplan–Meier survival estimates for matched cohort, by clinic type

Kaplan–Meier curves for the matched cohort show greater attrition (loss to follow-up or death) among men at general clinics, compared to male clinics (Fig. 2, see Supplementary file 2, Fig. 3 for Kaplan–Meier curves by clinic).

There was a small association between type of clinic and attrition [HR 0.71 (95% CI 0.60–0.85) for male clinic compared to matched patients at general clinics] (Table 3).

**Table 3:** Results from Cox regression models showing effect of male clinics (vs general clinics) on attrition

Variation #	Description	HR (95% CI)	Person-time (years)	Number of events
Main model	Primary analysis using propensity-score matched cohort, no covariates included	0.71 (0.60–0.85)	1451	506
1	Include only Male Clinic 1 and respective matched controls^a^	0.67 (0.53–0.85)	1085	289
2	Include only Male Clinic 2 and respective matched controls^a^	0.99 (0.75–1.31)	3957	195
3	Include only Male Clinic 1 and respective matched controls from first two years of clinic operating^b^	0.53 (0.34–0.82)	4362	109
4	Secondary definition of outcome^c^	0.83 (0.69–1.00)	1568	448
5	Not considered lost to follow-up if patient had viral load within 1 year	0.82 (0.68–0.99)	1568	431
Quantitative bias analysis of main model results (95% interval)				
	HR adjusted for systematic error	0.73 (0.71–0.78)	1451	506
	HR adjusted for systematic and random error	0.74 (0.61–0.89)	1451	506

^a^Not necessarily the same general clinic comparison group as in the main model, as propensity scores were generated again using only the included male clinic

^b^i.e. excluding all male clinic patients initiating ART after 1 June 2016 and their respective controls, and ending follow-up time for male clinic patients on 1 June 2016

^c^Loss to follow-up defined by clinic as missing an appointment by more than three months, with patient no longer considered lost to follow-up if they return to the clinic by the end of follow-up period

### Sensitivity Analyses

In separate analyses of male clinics compared to a newly matched cohort of general clinic patients, Male Clinic 1 showed a similar protective effect against attrition (HR 0.67; 95% CI 0.53–0.85), but no protective effect was observed for Male Clinic 2 (HR 0.99; 95% CI 0.75–1.31) (Table 3, for Kaplan–Meier curves see Fig. 4 in Supplementary file 2). Using the full matched cohort (both male clinics), a weaker protective effect was observed when changing the definition of loss to follow-up to a missed appointment with no return to the clinic by the end of follow-up period. Similar results were found reclassifying failures who had a viral load result within one year of their last visit.

### Quantitative Bias Analysis

Incorporating total error, the median adjusted HR was 0.74 (95% interval: 0.61–0.89) (Table 3). Adjusting for the hypothesized distributions of confounder prevalence in general clinics, confounder-male clinic associations, and confounder-attrition associations, there was still an small protective effect of male clinics on attrition (Supplementary file 1).

## Discussion

This study compared attrition from care in two male-targeted clinics and six general primary healthcare clinics in a low-resource, high HIV prevalence area. Males at the male-targeted clinics had a somewhat lower (HR 0.71; 95% CI 0.60–0.85) attrition than those in general clinics, adjusting for baseline clinical and demographic characteristics using a matched propensity score approach. Separate analyses of the two male clinics showed a protective effect only for Male Clinic 1 when compared to general clinics.

Men initiating ART at the male clinics were younger, with less advanced HIV than those initiating in the general primary healthcare clinics. This may be partly because, while not quantified in this paper, a large proportion of male clinic ART patients initially presented with STIs [44], which men may not be willing to reveal to female healthcare providers [64]. STI treatment is an important opportunity for HIV diagnosis and treatment initiation for males, who otherwise have fewer opportunities to interact with the healthcare system than women [65, 66], thereby making them less likely to be diagnosed and treated with HIV earlier in disease progression. Male clinics might also be more successful at linking newly diagnosed patients to ART care earlier in HIV disease progression. Earlier ART initiation also reduces the risk of transmission [35].

Despite being younger and healthier at ART initiation, male clinic patients (particularly those at Male Clinic 2) had a higher risk of 6- and 12-month attrition than general clinic patients in a crude analysis (Table 2). This is consistent with other literature showing that being younger and having fewer comorbidities makes patients more likely to drop out of care, possibly because they do not see an immediate benefit of ART [67, 68]. This is an important consideration when evaluating programs that improve uptake of testing or earlier ART initiation: attrition may appear to worsen because of changing baseline characteristics.

Through propensity score matching, we created a general clinic cohort that was more comparable to the male clinic group in terms of baseline health and demographic characteristics. In this analysis, we found a lower attrition from male clinics than from general clinics. When defining loss to follow-up as a gap in care with no return to clinic in the study period, the observed effect was less protective. This suggests that male clinics may be particularly protective against shorter disengagements from care, which is a growing concern with increasing ART duration [69–71]. It is possible that creating a male-only environment can make clinics more acceptable to men, possibly through helping them to overcome social and cultural barriers to care [38–40], including complicated gender dynamics with female healthcare providers [72], and leading to improved retention in care. Research has also suggested that men’s healthcare experiences are influenced by peers and social networks [73], so it is possible that the benefits of the service are enhanced through these relationships.

In an analysis of Male Clinic 2 compared to general clinics, no protective effect was observed. Although Male Clinic 2 only began ART services in mid-2016, Male Clinic 1’s lower attrition cannot be explained by the fact that the older clinic has had longer to establish itself, as protective effects were also observed in the first two years of Male Clinic 1’s ART services. It is possible that Male Clinic 2 attracted a more transient population as it is located at a transport hub, where men may have attended when convenient, as opposed to selecting a clinic near their homes. Most attrition happened soon after ART initiation. In a context where people have precarious livelihoods, with 38% unemployment in Khayelitsha, time and transport costs may present early barriers to continuing ART care [74, 75]. It is also possible that support from Médecins Sans Frontières via the lay adherence counselor provided at Male Clinic 1 differed from the standard of care at Male Clinic 2 and other clinics, where counselors were provided by another organisation and paid less than the Male Clinic 1 counselor. Nonetheless, Male Clinic 2 was successful in initiating younger, healthier men, with comparable retention to general clinics, when controlling for these factors. Despite favorable results compared to general clinics, there was 23% attrition by 12 months at Male Clinic 1. This likely reflects the myriad documented challenges to continued engagement in ART care including competing responsibilities, social stigma, mental health issues, migration and substance abuse [76–82].

There is a limited amount of literature on successful strategies to identify men living with HIV and link them to care [13]. Strategies include index testing, targeted community-based testing [83], self-testing for HIV [84], and incentives for ART initiation and retention [85]. This study adds important evidence about the type of clinical setting that can contribute to retaining males in HIV care.

Data for this analysis was limited to laboratory records and clinical data from selected clinics, and may have failed to detect deaths which actually occurred. In addition, data quality may differ between clinics, leading to differential misclassification of loss to follow-up. For multiple imputation, we assumed missing data was missing at random. However, the sensitivity analysis incorporating laboratory data to approximate “silent transfers” did not substantially change our results. A second limitation is the potential for unmeasured confounding, stemming from self-selection of males into the male clinics based on factors that were not measured in our limited set of potentially confounding covariates. Our quantitative bias analysis showed that even if there were reasonably strong confounder-exposure and confounder-attrition associations, there would still be a protective effect of male clinics on attrition. The hypothesized confounding characteristics in the male clinic patients may also increase the risk of attrition. For example, characteristics that are associated with a strong aversion to female healthcare workers may also be associated with other health behaviors such as avoidance of clinics in general, and non-disclosure of their HIV status, which is associated with poorer retention [86, 87]. If this is the case, then the observed effect sizes are underestimates and the true effect of male clinics may be more protective.

## Conclusions

In an environment where engaging health services may be perceived as a sign of vulnerability [88], contrary to hegemonic masculine ideals [89], these clinics aim to create explicitly male spaces to make men feel more comfortable attending the clinic and reduce attrition from care. Our results suggest this has been successful at Male Clinic 1. Both clinics successfully attracted younger, healthier males than the general clinics, reaching men sooner after HIV infection. Stand-alone male-only clinics may not always be feasible, particularly in lower-resource, or lower population density settings. However these findings support smaller-scale interventions such as increasing the proportion of male nurses and lay counselors in public clinics, or strengthening male-targeted STI treatment as an entry-point for males. Further research could explore other ways of designating male services such as separate spaces, rooms, programs, queues, entrances, or operating hours for men, which may also help to improve confidentiality and counter the perception of healthcare facilities as female spaces.

## Supplementary Information

Below is the link to the electronic supplementary material.

Supplementary file1 (DOCX 901 KB)

Supplementary file2 (DOCX 169 KB)

## Data Availability

The data used for this analysis is routine clinical data that cannot be passed on to third parties without prior research approval from the relevant health authorities (the City of Cape Town: https://www.capetown.gov.za/City-Connect/Access-information/Submit-a-research-request or Western Cape Provincial Department of Health: http://nhrd.hst.org.za/). The data is stored and managed by the Provincial Health Data Center^1^.

## Abbreviations

ART: Antiretroviral therapy
CD4: Cluster of differentiation 4
CI: Confidence interval
HIV: Human immodeficiency virus
HR: Hazards ratio
IQR: Interquartile range
STI: Sexually transmitted infection
WHO: World Health Organization

## Code Availability

Stata code for all analyses are available here: https://github.com/talicassidy/males.
