# Supplementary material for: Attrition from Care Among Men Initiating ART in Male-Only Clinics Compared with Men in General Primary Healthcare Clinics in Khayelitsha, South Africa: A Matched Propensity Score Analysis
Source: AIDS Behav. 2022 Jul 31;27(1):358–69. doi: 10.1007/s10461-022-03772-9 (PMC9852215; doi:10.1007/s10461-022-03772-9)
Supplement: Supplementary file 1 — Supplementary file1 (DOCX 901 KB) [file 10461_2022_3772_MOESM1_ESM.docx]

# Appendix 1: Quantitative Bias Analysis

## Methods

Unmeasured confounding could originate from residual confounding or from confounders that were not measured in the dataset and so cannot be controlled analytically. For example, males that choose to go to the male clinic may be more likely to be proactive in selecting a clinic of their choice, and therefore more engaged and more likely to stay in care. A hypothesized cluster of behavioural characteristics, including mental health and attitudes towards healthcare and female healthcare workers, may be associated with male clinic attendance and attrition. Considering this cluster of characteristics as a single confounder, we conducted a quantitative bias analysis.

In order to quantify the effects of this unmeasured confounding, we hypothesized a plausible distribution of associations between the confounder and male clinic attendance, and between the confounder and attrition at any time. As we were concerned that protective effects may be explained by uncontrolled confounding, we considered only associations that, if properly adjusted for, would reduce any observed protective effect of male clinics against attrition. We quantify the effect of this range of associations on the observed effect of male clinics on attrition.

We obtained a range of hypothesized associations between the confounder and attrition based on literature on risk factors for attrition, such as mental health, poverty, and substance use, which were not measured in our dataset. A meta-analysis of American studies found that patients with mental health diagnoses or symptoms had 6% lower retention (OR = 0.94; 95% CI = 0.90–0.99)[1]. A systematic review identified 52 studies of the association between ART adherence and depression, which found null effect sizes in 22 studies. Of the 18 studies that quantified the effects as risk ratios or odds ratios, 14 showed a one- to two-fold increase in poor adherence in those with diagnosed depression [2]. Another pooled meta-analysis of 11 studies conducted in sub-Saharan Africa found that good adherence was 55% lower among those with depression symptoms compared to those without (OR=0.45; 95% CI: 0.31–0.66). In the same review, a meta-analysis was not possible for five studies of the effects of alcohol use on adherence, which used heterogenous alcohol use measures and showed effects varying from harmful to positive[3]. We assumed that any cluster of confounding characteristics associated with male clinic attendance would not have a greater effect on attrition than depression does on adherence. However, it is possible that the confounder has a larger effect on retention than that of mental health diagnoses or symptoms in American HIV cohorts (pooled OR = 0.94) [1]. We therefore hypothesized that confounding characteristics associated with male clinic attendance and protective against attrition could lead to 0.5 to 0.9 times the risk of attrition, which we represent as a triangular distribution with a mode of 0.7.

We represented uncertainty in the confounder exposure association by specifying the confounder prevalence as a triangular distribution with a mode of 10% (range: 0-20%) in the general clinics. We assume a 1.5-2.5 times higher prevalence in the male clinics (triangular distribution, mode=2), resulting in a range of confounder prevalence of 0-50% in the male clinics. It is unlikely that the confounder is more than 2.5 times more prevalent in the male clinics: this is a low-income area and we can assume that other non-confounding factors inform clinic choice such as convenience and transport costs.

Using R [4], we ran a Monte Carlo simulation in which we sampled 100 000 draws from each of these three distributions: (1) confounder-attrition relative risk, (2) confounder prevalence in general clinics, and (3) confounder-male clinic relative risk. For each simulation, the sampled values from the three distributions were used to calculate four values: the probabilities of confounder status for each outcome and exposure status. These probabilities were used to randomly impute confounder status for each record, based on their observed exposure and outcome status, using a Bernoulli trial. We calculated the HR for each simulation, adjusting for the simulated confounder. Random error was sampled from a normal distribution (mean=0, SD=standard error of the unadjusted HR) and added to each adjusted HR. The resulting distribution of 100 000 HRs shows a range of results that incorporate (hypothesized) systematic and random error [5]. The median bias-adjusted HR is presented, along with a 95% interval (2.5-97.5 percentile).

In addition to this bias analysis, we also explored a range of possible associations (confounder-exposure and confounder-outcome risk ratios) which would nullify any observed exposure-outcome association. The e-value is the lowest strength of association risk ratio (equal for confounder-exposure and confounder-outcome) that would explain away an observed effect[6].

## Results

Figure 1 shows the distribution of selected parameters used in the quantitative bias analysis.


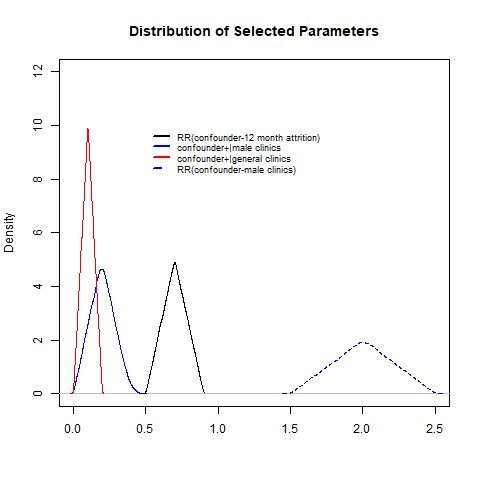


Figure 1 Distribution of simulated values of risk ratio (confounder-12 month attrition), and prevalence of the confounder in the male and general clinics

Figure 2 shows the HR estimate from the main analysis, as well as the adjusted HR from the quantitative bias analysis (see also Table 1). The black line shows a distribution of simulated HR values after adjusting for the hypothesized confounding using the distribution of parameters in Figure 1. The blue line shows the same simulated values, with random error added, approximating the total error. Incorporating total error, the median adjusted HR is 0.74 (95% interval: 0.61-0.89).


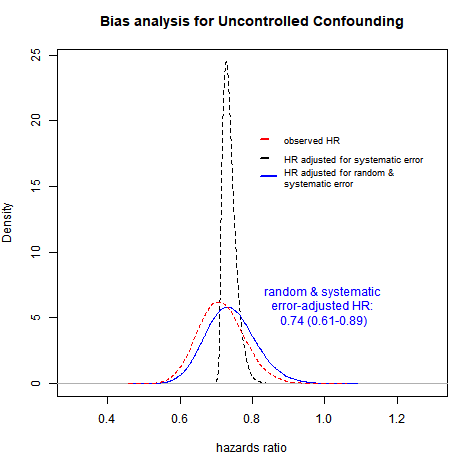


Figure 2 Distribution of estimated hazards ratio (per analysis above), the hazards ratio including simulated systematic error, and hazards ratio distribution incorporating random and systematic error

Table 1 Hazards ratios incorporating systematic and random error

| Description | HR | (95% Interval) |
| --- | --- | --- |
| Primary analysis using propensity-score matched cohort, no covariates included | 0.71 | (0.69-1.00) |
| HR adjusted for systematic error | 0.73 | (0.71-0.78) |
| HR adjusted for systematic and random error | 0.74 | (0.61-0.89) |

**References**

1. Rooks-Peck CR, Adegbite AH, Wichser ME, Ramshaw R, Mullins MM, Higa D, et al. Mental health and retention in HIV care: A systematic review and meta-analysis. Heal Psychol [Internet]. 2018 Jun;37(6):574–85. Available from: https://linkinghub.elsevier.com/retrieve/pii/S0031938416312148

2. Springer SA, Dushaj A, Azar MM. The impact of DSM-IV mental disorders on adherence to combination antiretroviral therapy among adult persons living with HIV/AIDS: A systematic review. AIDS Behav. 2012;16(8):2119–43.

3. Nakimuli-Mpungu E, Bass JK, Alexandre P, Mills EJ, Musisi S, Ram M, et al. Depression, alcohol use and adherence to antiretroviral therapy in sub-Saharan Africa: A systematic review. AIDS Behav. 2012;16(8):2101–8.

4. RCoreTeam. R: A language and environment for statistical computing. Vienna, Austria: R Foundation for Statistical Computing; 2014.

5. Lash TL, Fox MP, Fink AK. Applying quantitative bias analysis to epidemiologic data. Springer Science & Business Media; 2011.

6. Van Der Weele TJ, Ding P. Sensitivity analysis in observational research: Introducing the E-Value. Ann Intern Med. 2017;167(4):268–74.

7. Linden A, Mathur MB, VanderWeele TJ. EVALUE: Stata module for conducting sensitivity analyses for unmeasured confounding in observational studies. Stat Softw Components. 2019 Feb 16;

# R Code for quantitative bias analysis of unmeasured confounding

### Record level PBA unmeasured confounding

library("trapezoid")

library("ggplot2")

library(stringr)

library("survival")

## number of simulations

sims<-100000

## load dataset and generate observed cells

data<- read.csv("combined_bias_analysis.csv")

exp<-sum(data$maleclinic)

unexp<-sum(1-data$maleclinic)

n_total<-nrow(data)

unexp<-sum(1-data$maleclinic)

d<-sum(data$fail)

ud<-sum(1-data$fail)

n_total<-nrow(data)

# exp unexp

#D ac bc

#ud cc dc

#

ca<-sum((subset(data,data$fail==1))$maleclinic)

cc<-sum((subset(data,data$fail==0))$maleclinic)

cb<-sum(1-(subset(data,data$fail==1))$maleclinic)

cd<-sum(1-(subset(data,data$fail==0))$maleclinic)

## gen standard error

surv_object <- Surv(time = data$time, event = data$fail)

fit<-coxph(surv_object ~ data$maleclinic)

se<-(fit$var[1])^0.5

error<-rnorm(sims,mean=0, 1)*se

hr_crude<-exp(fit$coefficients[1])

## gen var for fail by 12 months

#data$fail12<-ifelse(data$time<365 & data$fail==1, 1, 0)

#Same result if just use fail:

data$fail12<-ifelse( data$fail==1, 1, 0)

x<-1

### risk ratio of confounder and disease

# distribution

rrcd<- rtrapezoid(sims, min = 0.5, mode1 =0.7, mode2 =0.7, max = 0.9)

##########P Prevalence of Confounder among unexposed and exposed

# distribution

prev_conf_unexp<- rtrapezoid(sims, min = 0, mode1 =0.1, mode2 =0.1, max = 0.2)

rr_con_exp<-rtrapezoid(sims, min = 1.5, mode1 =2, mode2 =2, max = 2.5)

prev_conf_exp<- prev_conf_unexp*rr_con_exp

### Note: each cell's value is a vector of probabilistically generated values ( N=sims),

#### which then generate 4 vectors (p1-4) of predictive values for confounder status based on disease and exposure status

exp_conf<-prev_conf_exp*(exp)

unexp_conf<- prev_conf_unexp*unexp

a1<-rrcd*exp_conf*ca/(rrcd*exp_conf+exp-exp_conf)

b1<-(rrcd*unexp_conf*cb)/(rrcd*unexp_conf+unexp-unexp_conf)

c1<-exp_conf-a1

d1<-unexp_conf-b1

p_conf_a<-a1/ca

p_conf_b<-b1/cb

p_conf_c<-c1/cc

p_conf_d<-d1/cd

##corrected HR vector

hr_corrected<-c()

#### begin sims* simulations of confounder distributions

for (i in 1:sims) {

## impute values of confounder for each record by selecting from bernoulli distribution

## the probability of confounder=1 for each record is informed by below forumula,based on outcome and exposure status of each record

data$conf<-rbinom(n_total,1,(p_conf_a[i]*(data$maleclinic*data$fail12)+p_conf_b[i]*(1-data$maleclinic)*(data$fail12)+p_conf_c[i]*(data$maleclinic)*(1-data$fail12)+p_conf_d[i]*(1-data$maleclinic)*(1-data$fail12) ))

#### calculate HR in simulated dataset, adjusting for confounder

surv_object <- Surv(time = data$time, event = data$fail)

fit<-coxph(surv_object ~ data$maleclinic+data$conf)

## Save HRs (also saves conf. HRs)

hr_corrected[i]<-exp(fit$coefficients[1])

fit<-coxph(surv_object ~ data$maleclinic)

# print remaining numbers of simulations to keep track of progress

print(sims-i)

}

## make vactors of Hazards ratios

hr_crude_se<-exp(log(hr_crude)-error)

hr_crude_list<-rep(hr_crude,sims)

hrtot <- exp(log(hr_corrected)-error)

### 95% intervals & medians of each HR vector

### observed

lower1<-round(quantile(hr_crude_se , probs = 0.025),2)

upper1<-round(quantile(hr_crude_se , probs = 0.975),2)

med1<-round(quantile(hr_crude_se , probs = 0.5),2)

lower1

upper1

med1

### adjusted - sys error

lower2<-round(quantile(hr_corrected , probs = 0.025),2)

upper2<-round(quantile(hr_corrected , probs = 0.975),2)

med2<-round(quantile(hr_corrected , probs = 0.5),2)

lower2

upper2

med2

### total

lower<-round(quantile(hrtot , probs = 0.025),2)

upper<-round(quantile(hrtot , probs = 0.975),2)

med<-round(quantile(hrtot , probs = 0.5),2)

#### PLOT RESULTS

bmp("HR_distributions_19nov2019.bmp")

plot(density(hr_corrected), main="Bias analysis for Uncontrolled Confounding", xlim=c(0.6,1.4), lty=2, xlab="hazards ratio")

lines(density(hrtot), col="blue")

lines(density(hr_crude_se), col="red", lty=2)

#abline(v=hr_crude,col="red", lty=4)

#abline(v=median(na.exclude(hr_corrected)),col="black", lty=3)

legend(1,12.5, c("observed HR","adjusted HR, systematic error", "adjusted HR, random & \nsystematic error"),lty=c(2,2,1),bty="n", lwd=c(2.5,2.5,2.5),col=c("red","black","blue"), cex=0.74) # gives the legend lines the correct color and width

text(1.1,5,"Adjusted HR: ",col="blue")

text(1.1,4.1,paste(med," (",lower,"-",upper,")",sep=""),col="blue")

dev.off()

###plot parameters

bmp("parameters.bmp") #, width = 1000, height = 1000)

#rrce<- prev_conf_exp/prev_conf_unexp

plot(density(rrcd), main="Distribution of Selected Parameters", xlim=c(0,2.5), xlab="",ylim=c(0,12))

lines(density(prev_conf_exp), col="blue")

lines(density(prev_conf_unexp),col="red")

lines(density(rr_con_exp), col="blue", lty=2)

#legend(0.5,10, c("RR(confounder-12 month attrition)", "confounder+|male clinics", "confounder+|general clinics"),lty=c(1,1,1),bty="n", lwd=c(2.5,2.5,2.5,2.5),col=c("black","blue","red", "blue"), cex=0.74)

legend(0.5,10, c("RR(confounder-12 month attrition)", "confounder+|male clinics", "confounder+|general clinics","RR(confounder-male clinics)"),lty=c(1,1,1,2),bty="n", lwd=c(2.5,2.5,2.5,2.5),col=c("black","blue","red", "blue"), cex=0.74)

dev.off()
