# Supplementary material for: Attrition from Care Among Men Initiating ART in Male-Only Clinics Compared with Men in General Primary Healthcare Clinics in Khayelitsha, South Africa: A Matched Propensity Score Analysis
Source: AIDS Behav. 2022 Jul 31;27(1):358–69. doi: 10.1007/s10461-022-03772-9 (PMC9852215; doi:10.1007/s10461-022-03772-9)
Supplement: Supplementary file 2 — Supplementary file2 (DOCX 169 KB) [file 10461_2022_3772_MOESM2_ESM.docx]

**Appendix 2: Extra tables and figures**


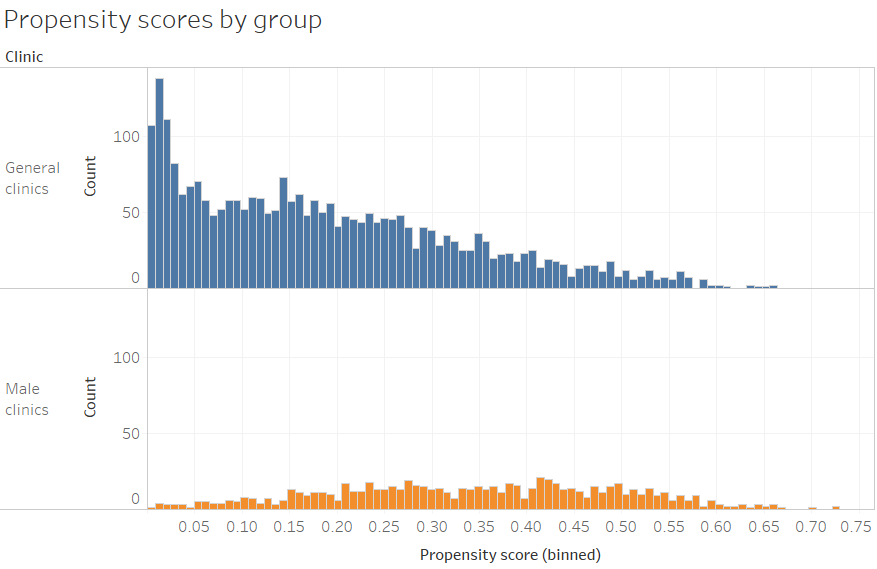


Figure 1 Distribution of propensity scores by clinic type before matching

Figure 1 shows a reasonable separation between general and male clinics, with many general clinic attendees having a low predicted probability of attending a male clinic. However, because of a larger sample of general clinic attendees, there was sufficient “common support”(1): there were enough general clinic patients with a similar range of propensity scores to male clinic patients to allow for close matching.

1. Thoemmes FJ, Kim ES. A Systematic Review of Propensity Score Methods in the Social Sciences. Multivariate Behav Res [Internet]. 2011 Feb 7;46(1):90–118. Available from: http://www.tandfonline.com/doi/abs/10.1080/00273171.2011.540475

Men in the male clinics and comparison groups were younger and healthier.

Figure 2 Comparison of characteristics of male clinic patients, and matched and unmatched general clinic male patients

The plot of the residuals over time (Figure 3) shows potential violations of the assumption of proportional hazards only after 2 years, where data is scarcer.

Figure 3 Plot of Schoenfeld residuals over time

Figure 4 Kaplan-Meier curves of Male Clinic 1 and Male Clinic 2 attrition compared to respective matched controls

Table 1 Survival (not deceased or lost to follow-up) at 6 and 12 months, by covariates

|  | Survival at 6 months^†^ | Survival at on year^†^ |
| --- | --- | --- |
| Total | 75% | 68% |
| Clinic |  |  |
| General Clinics | 72% | 63% |
| Male Clinic 1 | 82% | 78% |
| Male Clinic 2 | 72% | 63% |
| Initiating in year: |  |  |
| 2014 | 83% | 76% |
| 2015 | 80% | 74% |
| 2016 | 76% | 68% |
| 2017 | 71% | 63% |
| Guidelines CD4<350 (before 2015) | 83% | 76% |
| Guidelines CD4<500 (1 Jan 2015- 31 Aug 2016) | 80% | 73% |
| After Universal Test and Treat (1 Sept 2016) | 71% | 64% |
| Age |  |  |
| 18-25 years | 76% | 67% |
| 25-35 years | 74% | 67% |
| 35+ years | 75% | 69% |
| WHO stage at initiation |  |  |
| Stage 1 | 65% | 68% |
| Stage 2 | 74% | 62% |
| Stage 3 | 80% | 80% |
| Stage 4 | 25% | 25% |
| Stage 2-4 | 73% | 72% |
| Stage missing | 72% | 62% |
| Baseline CD4 Count |  |  |
| <200 | 76% | 69% |
| 200-350 | 77% | 69% |
| 350-500 | 75% | 69% |
| >500 | 73% | 63% |
| CD4 count missing | 72% | 60% |
| ^†^ calculated with life tables in Stata 15 using the primary outcome definition of attrition | | |

Table 2 Results of covariate adjustment of primary models showing adjusted hazards ratios and % change

| variable | Adjusted HR (clinic on attrition) | % change |
| --- | --- | --- |
| (crude HR: 0.71) | |  |
| WHO stage (continuous) | 0.715 | 0.02% |
| WHO stage (indicator vaiable) | 0.708 | 0.91% |
| WHO stage (stage 1 vs >1) | 0.714 | 0.13% |
| Baseline CD4 count | 0.715 | -0.03% |
| Baseline CD4 count (categorised) | 0.717 | -0.35% |
| Age (continuous) | 0.715 | -0.02% |
| Year of ART initiation (indicator variable) | 0.719 | -0.56% |
| Year of ART initiation (continuous) | 0.717 | -0.39% |
| Started ART Aug 2011-31 Dec 2014 | 0.713 | 0.29% |
| Started ART 1 Jan 2015 - 31 Aug 2016 | 0.716 | -0.13% |
| Started ART after 31 Aug 2016 | 0.712 | 0.35% |
| ART start date | 0.707 | 1.04% |
| Propensity score | 0.713 | 0.17% |

Table 3 Summary of Cox regression results with person time, events and goodness of fit test results

| Variation # | Description | HR  (95% CI) | Person-time males clinics (years) | Person-time general clinics(years) | Number of events, males clinics | Number of events, general clinics | Goodness of fit tests using Schoenfeld residuals (p-value) |
| --- | --- | --- | --- | --- | --- | --- | --- |
| Main model | Primary analysis using propensity-score matched cohort, no covariates included | 0.71 (0.60-0.85) | 735 | 716 | 215 | 291 | 0.953 |
| 1 | Include only Male Clinic 1 and respective matched controls | 0.67 (0.53-0.85) | 538 | 547 | 117 | 172 | 0.509 |
| 2 | Include only Male Clinic 2 and respective matched controls | 0.99 (0.75-1.31) | 197 | 198 | 98 | 97 | 0.206 |
| 3 | Secondary definition of outcome | 0.83  (0.69-1.00) | 147 | 289 | 30 | 79 | 0.876 |
| 4 | Not considered LTF if patient had viral load within 1 year | 0.82  (0.68-0.99) | 761 | 807 | 201 | 247 | 0.917 |

**Multiple imputation**

Table 4 Missingness and imputation of CD4 and WHO Stage data

| **Variable** | **Complete** | **Incomplete** | **Imputed** | **Total** |
| --- | --- | --- | --- | --- |
| WHO disease stage | 3205 | 305 | 305 | 3510 |
| CD4 count | 3089 | 421 | 421 | 3510 |

Table 5 Results from logistic regressions of predictors of missing CD4 count and Missing WHO stage data. Predictors shown were all included in Multiple imputation models

|  | Odds Ratio | P-value | [95% Conf. | Interval] |
| --- | --- | --- | --- | --- |
| Missing CD4 | | | | |
| Age at ART initiation | 1.01 | 0.007 | 1.003973 | 1.025825 |
| Time to attrition/censor | 1 | 1 | 0.9996445 | 1.000356 |
| LTFU/death | 1.22 | 0.131 | 0.9432783 | 1.572119 |
| Guidelines CD4<500 (1 Jan 2015- 31 Aug 2016) | 0.13 | <0.001 | 0.0794177 | 0.2275894 |
| After Universal Test and Treat (1 Sept 2016) | 0.56 | 0.159 | 0.2487528 | 1.256322 |
| Initiation date | 1.00 | 0.297 | 0.9996454 | 1.001164 |
| Male clinic | 0.51 | <0.001 | 0.3652777 | 0.7032761 |
| Missing WHO stage data | | | | |
| Age at ART initiation | 1.014 | 0.019 | 1.00245 | 1.027396 |
| Time to attrition/censor | 0.999 | 0.879 | 0.9994989 | 1.000429 |
| LTFU/death | 1.19 | 0.242 | 0.8878178 | 1.60259 |
| Guidelines CD4<500 (1 Jan 2015- 31 Aug 2016) | 0.48 | 0.012 | 0.2750085 | 0.8543364 |
| After Universal Test and Treat (1 Sept 2016) | 1.13 | 0.791 | 0.4567113 | 2.799218 |
| Initiation date | 1.00 | 0.372 | 1.00E+00 | 1.001242 |
| Male clinic | 0.39 | <0.001 | 0.2628442 | 0.5805669 |
